# A research study review of effectiveness of treatments for psychiatric conditions common to end-stage cancer patients: needs assessment for future research and an impassioned plea

**DOI:** 10.1186/s12888-018-1651-9

**Published:** 2018-04-03

**Authors:** Ralph J. Johnson

**Affiliations:** Departments of Myeloma, TMC Catholic Chaplain’s Corps, and Houston Hospice, University of Texas—MD Anderson Cancer Center, Unit 439, 1515 Holcombe Blvd, Houston, Texas 77030 USA

**Keywords:** Psychiatry, Psycho-oncology, End stage cancer care, Depression, Delirium, Anxiety, Adjustment disorder, Literature review, Research needs assessment

## Abstract

**Background:**

Rates of psychiatric conditions common to end-stage cancer patients (delirium, depression, anxiety disorders) remain unchanged. However, patient numbers have increased as the population has aged; indeed, cancer is a chief cause of mortality and morbidity in older populations. Effectiveness of psychiatric interventions and research to evaluate, inform, and improve interventions is critical to these patients’ care. This article’s intent is to report results from a recent review study on the effectiveness of interventions for psychiatric conditions common to end-stage cancer patients; the review study assessed the state of research regarding treatment effectiveness. Unlike previous review studies, this one included non-traditional/alternative therapies and spirituality interventions that have undergone scientific inquiry.

**Methods:**

A five-phase systematic strategy and a theoretic grounded iterative methodology were used to identify studies for inclusion and to craft an integrated, synthesized, comprehensive, and reasonably current end-product.

**Results:**

Psychiatric medication therapies undoubtedly are the most powerful treatments. Among them, the most effective (i.e., “best practices benchmarks”) are: (1) for delirium, typical antipsychotics—though there is no difference between typical vs. atypical and other antipsychotics, except for different side-effect profiles, (2) for depression, if patient life expectancy is ≥4–6 weeks, then a selective serotonin reuptake inhibitor (SSRI), and if < 3 weeks, then psychostimulants or ketamine, and these generally are useful anytime in the cancer disease course, and (3) for anxiety disorders, bio-diazepams (BDZs) are most used and most effective. A universal consensus suggests that psychosocial (i.e., talk) therapy and spirituality interventions fortify the therapeutic alliance and psychiatric medication protocols. However, trial studies have had mixed results regarding effectiveness in reducing psychiatric symptoms, even for touted psychotherapies.

**Conclusions:**

This study’s findings prompted a testable linear conceptual model of co-factors and their importance for providing effective psychiatric care for end-stage cancer patients. The complicated and tricky part is negotiating patients’ diagnoses while articulating internal intricacies within and between each of the model’s co-factors. There is a relative absence of scientifically derived information and need for more large-scale, diverse scientific inquiry. Thus, this article is an impassioned plea for accelerated study and better care for end-stage cancer patients’ psychiatric conditions.

## Background

**Fig. 1:**
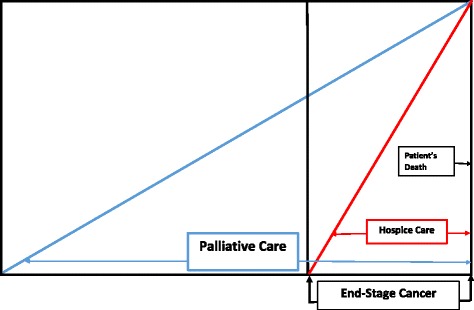
End stage cancer in relation to palliative and hospice care

Not surprisingly, psychiatric symptoms, disorders, and emotional distress are relatively common among cancer patients, in particular those with end-stage cancer ([1], also see [2]; see Fig. 1), which is the terminal phase of cancer—including hospice and the latter part of palliative care [1–29]).

The psychiatric symptoms and disorders in end-stage cancer patients have remained unchanged over time and include delirium at 20–65%, depression at 21%, and anxiety and adjustment disorder at 14% ([30–42], also see [4, 5, 7–9, 16, 19]). Note that this includes approximately 3% of end-stage cancer patients who also suffer from severe and persistent mental illnesses (e.g., schizophrenia, bi-polar disease, major depressive disorder) ([43], also see [4]). Though the rates have been consistent over time, the actual volume has increased due to the growing number of older adults in the general population and cancer within these populations being a chief cause of morbidity and mortality ([44, 45], also see [7]). Patients 65 years old and older are the fastest growing segment of the population and the incidence and prevalence of cancer and related psychiatric disorders among them is consequently expanding. [45] This is evidenced by the substantial and statistically significant increments in dispensing all classes of psychotropic medications in recent years among end-stage cancer patients. [46–49]

Psychiatric symptoms and disorders not only cause extreme suffering in their own right but can also exacerbate physical ailments and substantially degrade the quality of life, which end-stage cancer care aims to ameliorate ([50–52], also see [28]). In response to this growing need, psychiatric therapeutic interventions including psychopharmacology and psychosocial therapy alone or in combination have been used with end-stage cancer patients to treat commonly occurring psychiatric disorders (cf. [53], see [54], cf. [55], see [56], cf. [57], see [58], also see [6, 19, 23]).

Thus, treating psychiatric conditions in end-stage cancer patients is reaching a watershed in terms of both practice and research opportunities to assess treatment effectiveness (cf [46]). Key to addressing end-stage patients’ psychiatric symptoms and disorders, especially going forward, is the effectiveness of psychiatric interventions and the state of research assessing that effectiveness (cf [4, 18, 19, 23, 29]). Past literature reviews ([4], also see [7, 19]) on end-stage cancer patients’ psychiatric symptoms and disorders have included particular aspects of psychiatric treatment effectiveness—one of which is an ongoing and expanding internet compilation solely on depression [23]. Few, if any, studies provide a reasonably current comprehensive overview regarding the effectiveness of various psychiatric treatments with an assessment of the state of research and the need for further research. The intent of this article is to report on a recent systematic literature review study regarding the effectiveness of interventions for psychiatric conditions commonly prevalent among end-stage cancer patients; the review study assessed the state of the research into treatment effectiveness. Furthermore, the review study attempted to include non-traditional (i.e., alternative) therapies that have undergone scientific study regarding their effectiveness in treating end-stage cancer patients’ psychiatric symptoms and disorders. This has not been done previously. This article is intended to provide a well-integrated compendium of “pearls” (i.e., a psychiatric pharmacopeia). The goal is to inform oncologists and mental health practitioners regarding how to reduce end-stage cancer patients’ emotional distress and psychiatric discomfort and improve their quality of life—the ultimate goal of end-stage cancer care [1, 2, 11, 49].

## Methods

**Fig. 2:**
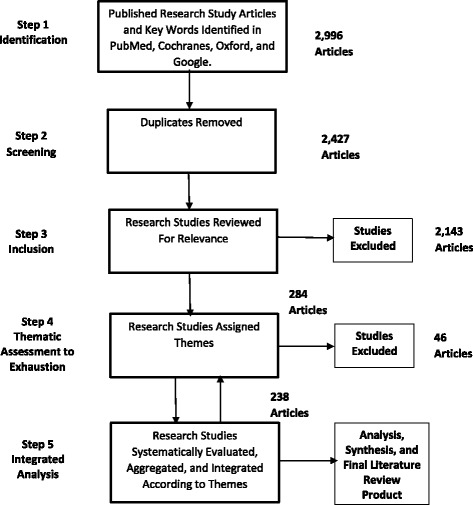
Research article collection and analysis flow diagram

A five-phase systematic strategy was used to derive the articles for this literature review, as illustrated in Fig. 2.

An initial PubMed search was undertaken to identify related articles. This also was used to identify and expand the keywords and keyword combinations (i.e., Booleans) in a second and more comprehensive search of the PubMed, Medscape, Cochranes, and Oxford databases. Studies that did not refer to psychiatry, end-stage cancer, or effectiveness of treatment were excluded. All studies, abstracts, reviews, raw reports, observational studies, and (random) controlled clinical trials found relevant have been included. A grounded [59–61] qualitative methodology was used to iteratively generate and assign themes to research study articles (i.e., code) until thematic saturation, an exhaustion of themes, was achieved. The inherent risk in this process is that some studies or themes will be missed, but the assumption is that a general and credible assessment will be produced [61]. The ultimate aim of this process was to produce an integrated, synthesized end-product consisting of brief descriptive accounts of the effectiveness of psychiatric interventions and a needs assessment on the state-of-effectiveness research.

## Results

Findings are organized in accordance with the prevalence of common psychiatric disorders found among end-stage cancer patients, the most employed treatments (i.e., the “best practices benchmarks”) (cf. [4, 18, 19, 23, 27, 43, 52]) followed by other treatments, and the state of research on their effectiveness, with recommendations for future inquiry.

### Delirium

Delirium is the most prevalent neuropsychiatric disorder in end-stage cancer patients ([62–64], also [7, 13, 25, 46, 62]). Antipsychotics (e.g., haloperidol, clorapromazide, methotrimeprazine, olanzapine, risperidone, quentiapine) are the mainstays for effective psychiatric symptom management of delirium ([65–69], also see [7, 13, 18]).

#### Typical (traditional) antipsychotics

Haloperidol has been and continues to be the psychiatric medication of choice and the best practices benchmark for end-stage cancer delirium symptom relief ([70–72], also see [7, 18, 19, 25, 46, 73]) It has low dose tolerance, flexibility in route administration (e.g., by mouth, intravenous, intramuscular, subcutaneous), relative safety, and high efficacy ([74–78], also see [7, 13, 18, 62, 65–67, 70]). Haloperidol and other typical antipsychotics (e.g., methotrine, epramazine) have also been reported to have additional analgesic properties. Evidence also suggests they are adjuncts to pain management or have anti-nausea/emetic effects [79–83]—especially for intractable nausea [84]. In addition, they have a controversial and ethically questionable role in terminal sedation, namely, rendering unconscious the end-stage patient who has truly distressing, hyper-aroused, and refractory delirium symptoms ([85, 86], also see [13]). Note that there are no official guidelines for practitioners and only limited data on the use of antipsychotics for terminal sedation [13, 87]. Antipsychotics may also be used in combination with lorazepam for rapid onset and augmentation of sedative effects in pronounced hyper-aroused delirious patients [7]. Reports have suggested that aripiprazole is effective with hypoactive delirium; however, it is ineffective with hyperactive delirium and may even trigger it. Chlopromazine has calmative effects in agitated or combative delirium patients [88], also see [19, 73].

#### Atypical (new-generation) antipsychotics

In studies and case reports, risperidone, clozapine, quetiapine, and levomepromazine have proven effective in low doses compared to traditional/typical antipsychotics but with reduced extra pyramidal symptom side-effects, which is the case in end-stage cancer patients ([89–92], also see [13, 14, 18, 65, 67, 88]). They also may have hypotensive and cardiac side-effects (e.g., arrhythmias), which must be monitored ([93–98], also see [7, 13, 18, 19, 36]). Like haloperidol, new-generation anti-psychotics can be used parenterally, which is advantageous with patients in end-stage cancer where oral medications are not possible [7, 18, 54, 72, 94]. These agents may have other side-effects such as hyperglycemia, hyperlipidemia, and weight gain, which must be reasonably balanced in light of the state of advancement in the end-stage cancer and symptom relief.([99–101], also see [7, 18, 19]).

Antipsychotics, whether traditional/typical or atypical/new-generation, are the approved psychiatric medications for patients with severe mental disorders who suffer from schizophrenia and in some cases bi-polar disorders and who also have end-stage cancer [29, 43].

#### State of antipsychotic scientific research for delirium symptom relief for end-stage cancer patients

To date, except for treating hyper-aroused/distressed delirium and particular medication side-effect profiles, reports in the literature have shown no difference between typical/traditional vs. atypical/new-generation antipsychotics in terms of effectiveness for delirium symptom reduction in end-stage cancer patients ([102], also see [7, 18, 19, 69, 96]). Thekdi, Trinidad, and Roth [19] noted that a small study found that advanced prophylactic use of antipsychotic medications in patients at risk for delirium reduced symptom occurrence later on in 50% of the cases. They further argued for early and aggressive use of low-dose antipsychotics with cancer populations with an anticipated trajectory toward end-stage disease. They also noted the absence of and, thus, called for definitive trial studies on the effectiveness of the newer generation of antipsychotic agents for cancer patients experiencing delirium, in particular end-stage cancer patients (e.g., asenapine, iloperidone, lorasidone, paliperidone) and others in the pipeline—about which for end-stage cancer patients almost nothing is known.

Masman ([103–106], also see [74]) reported that far more studies have been conducted on the traditional/typical as opposed to the atypical/new generation as the former have been around longer. Nevertheless, most studies are retrospective studies, overviews, or case reports and few have been prospective, large-scale trials, much less randomized controlled trials, regardless of old or new generation [106]. Also there is a tendency to echo shaky previous findings. Thus, critics of the research on antipsychotic treatments for delirium in end-stage cancer patients contend that most of it is the consequence of extrapolation and expert opinion (cf. [74, 103–106]). Given that one antipsychotic is as effective as any other except for side-effect profiles, there is definitely a need for solid large-scale prospective studies that also take into account patient type and type of delirium (e.g., hypo- vs. hyper-aroused) [18, 102]. Also, more research is needed to support and advance effective therapies for prevention and management of delirium, especially prospective random controlled clinical trials, that account for persistent, refractory symptoms and cumulative antipsychotic use in end-stage cancer patients. In sum, it appears that no large-scale prospective random controlled clinical trials have focused on antipsychotic treatments of delirium in end-stage disease, much less end-stage cancer [52].

### Clinical depressive disorder

Among end-stage cancer patients, depression is the second most common psychiatric disorder encountered ([107–109], also see [4, 9, 13, 18, 19, 23, 47, 53]).

#### Selective serotonin reuptake inhibitors (SSRIs)

The approach to treatment of depression depends on the patient’s life expectancy ([109], also see [110, 111]). If the patient’s life expectancy is 4–6 weeks or longer, then serotonin reuptake inhibitor **(**SSRI) antidepressants (e.g., escitalopram, citalopram, sertraline, fluoxetine, paroxetine, mirtazapine ([112, 113], also see [114]) are the medication of choice and the best practices benchmarks for effective treatment ([115], also see [4, 7, 18, 23, 25, 53, 73]). For end-stage cancer patients, these medications are relatively safe, inexpensive, and useful with co-morbid conditions like anxiety and irritability; they also serve as possible analgesic synergists ([116], also see [13, 18, 19]). One downside is that they take 4–6 weeks or more to titrate to a normal response dose to achieve a beneficial effect ([117, 118], also see [7, 13, 18, 19, 23, 114]). Reports have suggested that they have some side-effects (e.g., restlessness, agitation, insomnia, sedation, parkinsonianism) that can be problematic with end-stage cancer patients, especially with co-morbid delirium [7, 23]. On the other hand, compared to other classes of antidepressants, they have few interactions with other drugs [7, 13, 18, 19, 23, 119], which is an important consideration with end-stage cancer patients ([117, 118, 120], cf. [72], also see [21]). (Note: Kiener, Weixler, Massel, Gartner, et al. [121] revealed that end-stage cancer patients were taking an average of 4–6 different prescription medications and up to 11 at the point of death.)

#### Serotonin–norepinephrine reuptake inhibitors (SNRIs)

Serotonin–norepinephrine reuptake inhibitors (SNRIs; e.g., veralfaxine, duloxetine) like SSRIs are well tolerated and have similar side-effects, except that SNRIs lead to hypertension and have been reported to cause bleeding in rare cases [13]. In addition to the side-effects, their downside is that they may take substantially longer than SSRIs to achieve optimal effect (e.g., > 4–6 weeks) and end-stage cancer patients may not have that much time left. [4, 13, 18, 19, 23, 53, 114, 122].

#### Tricyclic antidepressants

Tricyclic antidepressants (TCAs; e.g., amoxapine, desipramine, imipramine, doxepin, clomipramine, amitriptyline, nortriptyline) are not well tolerated by end-stage cancer patients and have major anti-histaminic and anti-cholonergic side-effects (e.g., delirium, confusion, hallucinations); they also have the potential for serious drug-drug interactions—though some reports suggest they have analgesic effects ([4, 7, 13, 18, 53, 114, 122], cf. [19, 23, 25, 73]).

#### Bupropione

Bupropione is a well-tolerated antidepressant in end-stage cancer patients, and it triggers mild stimulation as a side-benefit in patients with chronic fatigue ([123], also see [13, 18, 19, 53]). However, for the same beneficial reasons, it is counter-indicated for patients prone to seizure disorders ([13, 19, 123], also see [53, 114]). Also, it might increase anxiousness, restlessness, and angina [123].

#### Mitrazapine

Mitrazapine was shown to be statistically significantly effective in that it not only improved depression symptoms over other available antidepressants but it also helped with anorexia, anxiety, and insomnia, though it led to constipation ([124, 125] also see [4, 19, 53]). In a small random controlled clinical trial, Cankurtaran, Ozalp, Soygur, Akbiyik, et al. [126] found that mirtazapine was more effective than imipramine in cancer patients in reducing depressive and adjunct symptoms.

#### Trazadone

Trazadone is considered too sedating and needs extremely high and potentially risky doses to be effective with end-stage cancer patients [7].

#### Monoamine oxidase inhibitors

Monoamine oxidase inhibitors (MAOs) are considered the least effective of antidepression medications for cancer patients in general, much less end-stage cancer patients [7, 23]. Thus, MAOs have been abandoned in favor of SSRIs [7]. MAOs also have a high number of drug-drug interactions and may lead to hypertensive crises with the wrong foods or medications [7, 23].

#### Ketamine

If the patient’s life expectancy is < 2–3 weeks, then an atypical psychiatric medication option to very effectively treat depression might be ketamine [127]. Ketamine has been studied in limited Phase I trials to treat refractory depression with a statistically significant 93% improvement rate within 0–3 days and an 80% post-dose improvement rate [127–129]. A statistically significant number of subjects showed few or no side-effects related to the medication [129]. A meta-analytic study of ketamine use in general palliative care found that it can be extremely effective in patients with refractory depression, especially those with chronic pain ([130–139], also see [5, 23]). However, it is unclear exactly for which patients ketamine is optimal or what the best modes for administration are.

#### State of antidepressant scientific research for clinical depression symptoms relief for end-stage cancer patients

Except for the trial for mitrazapine, ketamine, and fluxoetine [23, 140, 122, 126], few large-scale, prospective, systematic, rigorous, scientific randomized controlled clinical trials are reported in the literature on medication effectiveness in end-stage cancer patients ([141–145] also see [58, 140, 122, 126]). Much of what is known or suspected is based heavily on expert opinion extrapolated from case reports and/or small non-end-stage cancer patient studies [58, 114]. What has been clearly established regarding the efficacy of antidepressants in treating cancer patients, including end-stage cancer patients, is that an antidepressant—any antidepressant—is better than none at all—provided the patient has sufficient life expectancy ([146–148], also see [5, 7, 13, 18, 19, 23, 25, 53, 58, 73, 135, 141, 145]). That is, any antidepressant is as effective as another, except when considering their different side-effect profiles, potential for overdose, and concomitant drug-drug interactions.

End-stage cancer patient guidelines for antidepressants based on practitioners’ experience, expert opinion, case reports, the few small trials, and extrapolation appear to have been codified as scientific fact through a process of successive repetition and echoing in medical practice journals and then translated into best practices ([146–159, 160], also see [18, 19, 25, 114, 74]). Given that the discipline has recognized a paucity of solid scientific research, researchers have called for closer, more thorough, and more comprehensive examinations of the effectiveness of antidepressants for end-stage cancer patients suffering depressive symptoms ([152, 153], also see [52]). This is especially the case in the context of ethical considerations surrounding end-stage cancer patients’ refusal of medical care [52]. These calls have included the desperate need for large-scale, prospective, multi-site studies of end-stage histolologically similar cases [58]. Unfortunately, yet ultimately, the use of particular antidepressants with end-stage cancer patients suffering depression depends on practitioners’ preferences and individual preferences (e.g., side-effects, tolerance, poly pharmacy, reactions). As Fitzgerald, Lo, Li, Gagliese, et al. [154] noted, vital research is desperately needed to better understand treatment effectiveness in terms of the phenomenology of “subthreshold” depression as well as all aspects of depression in end-stage cancer patients to strengthen the evidence-based effectiveness of psychiatric best practice guidelines.

#### Psychostimulants

The other treatment option for depressive symptoms in end-stage cancer patients with a short (e.g., 2–3 weeks) life expectancy is low-dose psychostimulants (e.g., methylphenidate, dextro-amphetamine, modofinil) ([156, 157], also see [4, 5, 7, 13, 18, 19, 23, 25, 53, 73]). Low-dose psychostimulants have almost immediate benefit—peaking within 3–8 h ([158–174], also see [7, 13, 19, 23, 53]). Patients experience a marked elevation in mood, self-esteem, alertness, focus, cognitive function, and regulation of appetite [7, 13, 18, 19, 23, 53, 158]. A study of patients taking 10 mg methylphenidate twice a day, limited to 80 mg per day or adequate response, found that 23 out of 30 (77%) end-stage cancer patients treated had moderate to marked improvement in depressive symptoms ([167], also see [166]). Only two patients withdrew due to intractable side-effects ([167], also see [53]). In a similar study, 30 out of 41 patients (73%) showed improvement in 7 days [167].

Psychostimulants also are particularly effective in combating opiate sedation and synergizing analgesics [7]. Due to the immediacy of onset, there is no question regarding effectiveness and, thus, they are the treatment of choice and the best practices benchmarks for short-term care of end-stage cancer patients’ depression; they are often combined with SSRIs [4]. Note that there is a black box warning about methylphenidate causing cardiac arrest and mandating consent to use ([168], also see [13, 114]). Methylphenidate is also associated with side-effects such as headaches, anxiety, hypertension, and cardiac arrhythmias [23]. Additionally, modofinil has sympathomimetic effects and, thus, is a good choice for older end-stage cancer patients [7, 13, 114, 166, 168].

#### State of psychostimulant scientific research for clinical depression symptom relief for end-stage cancer patients

The effectiveness of psychostimulants in treatment of depression symptoms in cancer patients in general and (in the short term) end-stage cancer patients is fairly well established based on a series of scientifically sound randomized controlled clinical trials, the “gold standard” in research [4, 13, 18, 19, 74]. Nevertheless, and ironically, the use of these medications, though common in this population, may still be off-label (cf. [4, 74]).

### Non-traditional/alternative psychiatric therapies

#### Electroconvulsive treatment

Electroconvulsive treatment (ECT) is extremely effective in ameliorating depression and is remarkably safe in elderly cancer patients except those prone to seizures and cardiac problems ([54], also see [7]). ECT is especially effective in depression refractory to antidepressant medications. It does cause short-term memory loss, but cognitive effects are lower with unilateral as opposed to bi-lateral ECT. However, Winnell and Roth [7] noted ethical questions about the image of electrically shocking terminal cancer patients, especially elderly patients.

#### Herbal remedies

In a double-blind, cross-over randomized controlled clinical trial examining the effectiveness of guariana (paullina aipana) on 36 patients with breast cancer, despite guariana’s psychostimulant properties, no difference was detected between those receiving it versus those receiving a placebo [169, 160]. A review of studies on visum album L (European mistletoe) used in cancer patients who also suffered from depression and anxiety found that the treatment was effective for both depression and anxiety; [170, 172] mistletoe treatments were well tolerated with fewer side-effects than conventional treatments.

### State of non-standard/alternative psychiatric therapy scientific research for clinical depression symptom relief for end-stage cancer patients

Notably, Breitbart and Dickerman [23] claimed that of all the psychiatric therapies that treat depression, ECT‘s effectiveness has received the most definitive, extensive, solid, and generalizable scientific examination. Non-standard/alternative herbal studies, though few, seem to be well designed or account for drug-drug interactions well. They are proliferating outside the United States, with the particular herb studied indigenous to the cultural locality and region where found.

#### Adjustment disorder/anxiety

Adjustment disorder and anxiety are the third most common psychiatric conditions encountered with end-stage cancer patients. Moivic [4] reported that “according to the DSM-V ([175], also see [15, 57]). Adjustment Disorders are emotional/behavioral symptoms in excess of what would be normal response to a given stressor in excess of a mere diagnosis of depression” ([176], also see [4]). Since psychiatric medication interventions are similar to those for anxiety, they are included with anxiety, though the symptoms dictate treatment, and these interventions may also have depressive features [4].

End-stage cancer patients naturally experience anxiety and anxiety-related psychiatric symptoms and disorders, especially at crisis points ([7], cf. [15]). This can be considered normal people reacting normally to extremely abnormal circumstances. Determining the point at which anxiety becomes abnormal and pathological is tricky, making exactly when a therapeutic intervention is necessary problematic ([177–179], also see [7, 15, 25, 56]), cf. [180]. Also, anxiety can be a component of delirium or depression, as well as a pathological feature of the cancer itself or its treatment [179]. For example, dexamethasone and metoclorapromide can cause anxiety and restlessness along with emotional distress and hyperactivity [25].

Nevertheless, biodiazepines (BDZs—temazepam, midazolam, alopazalam, lorazepam, oxezapam, diazepam, clonazepam) are the most effective and powerful drugs in treating anxiety and related adjustment disorders, though they do cause sedation, confusion, motor confusion, and un-coordination, build up over time, inordinately affect elderly patients, pose a risk for renal dysfunction, and are associated with drug-drug reactions ([181, 182], also see [4, 7, 13, 18, 19, 74]). Also, there is an exception to their use with lung cancer patients in that the disease is associated with respiratory distress (i.e., air hunger) in patients and BDZs’ depressive effects can exacerbate this condition and increase related anxiety [183]. Similarly, caution must be used with BDZs as they are synergists with opiates, which can lead to deadly pulmonary depression [7, 13, 18, 19, 74]. Nevertheless, several studies have found that BDZs are effective and powerful in relieving anxiety and are adjuncts to relieving the pain, nausea, and emesis that end-stage cancer patients commonly suffer; BDZs can even help with end-stage sedation ([175, 184–192], also see [13, 85]) (Note: In lieu of BDZs, other medications that can be employed but are less effective are antidepressants, bupropione, and low-dose antipsychotics [7]). Whatever the case, Dauchy, Dolbeault, and Reich [114] cautioned that BDZs should not be prescribed indiscriminately for anxiety or depression because they might further complicate an already complicated clinical picture.

#### Nabalone

Maida, Ennis, Irani, Corbo, et al. [193], in a prospective study of nabalone (Casamet)—a cannabinoid—therapy in managing pain found a statistically significant (*P* = .0284) reduction in anxiety post-nabalone administration; nabalone also lowered or eliminated the use of anti-inflammatories.

#### Homeopathy

In a study on homeopathy and anxiety and anxiety disorders, Pilkington, Kirkwood, Rampes, Fisher, et al. [194] found that homeopathy was frequently and preferably used by a range of cancer patients suffering anxiety. Homeopathy was shown to be effective without side-effects, but the exact degree of effectiveness was questionable and needs further study.

#### Herbal remedies

Su, Wang, Grant, and Liu’s [195] comprehensive review of Chinese herbal medication trials with a range of cancer patients found that Chinese herbal remedies were ineffective in relieving anxiety. The exact herbal medicines were unclear. Also, though adverse events were related to the medicines, no serious adverse events were reported. A review of studies on visum album L (European mistletoe) found that herbal medicine was effective for both psychiatric disorders (cf. [196], also see [171, 172, 180]) and the treatments were well tolerated with fewer side-effects than conventional treatments.

### State of psychiatric therapy scientific research for adjustment disorder/anxiety relief for end-stage cancer patients

Research studies on BDZ interventions with end-stage cancer patients appear relatively robust and comprehensive. One reason offered for this is the overlap between BDZs and anxiety and the proliferation of post-traumatic stress disorder (PTSD) studies, extensions and extrapolations of those PTSD case and observational studies, meta-analytic reviews, and trials [4, 197–199]. Nevertheless, appearances might be deceiving in that Masman, van Dijk, Tibboel, Baar, et al. ([74], also see [200–202]), in their study on midozolam—one of the most prescribed BDZs in hospice settings—found that only case studies and small outdated trials were available to justify this practice in best practices guidelines (cf. [49]). Whatever the case, at a minimum, more and larger trials are needed on BDZs’ effectiveness to establish best practices guidelines, especially in conforming prescription of these drugs to the particular needs of end-stage cancer patients [74].

#### Psychosocial therapy

Many reports in the literature address psychosocial (i.e., talk) therapy interventions with communicative and cognizant end-stage cancer patients in terms of relieving psychiatric symptoms and emotional distress, including but not limited to individual counseling, hypnotherapy, group therapy, psychotherapy, cognitive behavioral therapy (e.g., relaxation training, biofeedback), dignity therapy, and existential therapy ([203–209], also see [4, 5, 17, 23–25, 52, 73, 114, 90]). The general consensus in the discipline is that psychosocial interventions can reduce psychiatric disorders in communicative and cognizant end-stage cancer patients. ([210, 211], also see [4, 17, 23, 114]).

#### Meaning-centered and dignity therapy

Both meaning-centered and dignity therapy have been tested in Phase I, multi-site, randomized controlled clinical trials with promising but mixed results in terms of efficacy with end-stage cancer patients ([210–219], also see [5, 17, 23, 52, 63, 203, 204]). Dignity therapy (i.e., therapy intended to enhance diminished dignity and achieve a sense of purpose) was related to higher levels of helpfulness, quality of life, and sense of dignity in end-stage cancer patients [52, 215]. However, the patients did not experience a reduction in emotional distress [219].

Meaning-centered psychotherapy refers to the brief psychotherapeutic intervention predicated in Victor Frankl’s logotherapy ([220], also see [52]). It aims to enhance psychosocial well-being in end-stage patients through individual and group applications [52, 203]. In a Phase I randomized controlled clinical trial, end-stage cancer patient study participants received group and individual psychotherapy; in a follow-up trial, individual psychotherapy was compared with a placebo intervention [220]. In both trials, participants receiving individual psychotherapy reported improvements in well-being and quality of life. However, the half-life of the intervention was brief in that differences in treatment outcomes were completely absent 2 months later (also see [52]).

#### Hypnotherapy

A small trial examining a hypnotherapy intervention designed to reduce anxiety for patients at an in-patient hospice facility found a statistically significant reduction in anxiety by the fourth treatment session ([221, 222], also see [52]).

#### Self-help

In a random controlled clinical trial, a brief guided self-help intervention was conducted with hospice in-patients targeting their depression symptoms ([223], also [52], cf. [114]). The intervention consisted of a trainer training participants to break cyclic cognitions of worry followed by practice sessions over a four-week period; in contrast, the control group received no intervention but crossed over in 4 weeks to the training intervention to receive treatment ([223], also see [52]). Participants experienced statistically significant reductions in anxiety but no reductions in depression ([223], also see [52]).

#### Orientation and cognitive behavioral therapy

Two other trials assessed the effectiveness of psychosocial interventions in cancer patients suffering from major depression. Both trials were enhanced orientation programs with the control group receiving the standard of care and both found reductions in depression symptoms ([224–226], also see [58], cf. [114]). Two other studies comparing the effectiveness of classic psychotherapy and cognitive behavioral therapy (CBT) found no reductions in depressive symptoms [58]. In a trial with cancer patients, Chen, Chen, and Zhi [227] found no difference between CBT and busprione and sertraline in terms of reducing depression and anxiety. They inferred that CBT was just as effective as psychiatric medication in treating anxiety and depression.

#### Psychosocial education

Between 1999 and 2002, a series of meta-analytic studies was conducted on previous studies (1970–1990) that examined the effectiveness of psychosocial education on emotional distress in cancer patients with respect to reducing depression and anxiety ([150], also see [114]). The studies universally found substantial evidence of a positive benefit on depression and anxiety in end-stage adult cancer patients but no difference in treatment effectiveness, especially in advanced disease cancer patients—even when delivered by experienced practitioners ([228–230], also see [59], cf. [114]). Notably, the investigators concluded that the interventions were beneficially effective in reducing emotional distress (also see [151]). Put differently, end-stage cancer patients appreciate one-to-one therapeutically delivered management of expectations and this type of intervention may be supportive of other interventions (also see [231]).

### State of psychosocial therapy scientific research for end-stage cancer patients’ psychiatric disorders

Other than the trials previously mentioned, no other reports were found that approximated large-scale, prospective, rigorous scientific inquiry regarding the effectiveness of psychosocial intervention with end-stage cancer patients’ psychiatric disorders. Nevertheless, the fact that psychosocial (i.e., talk) therapy is venturing into clinical trials to scientifically verify interventions’ effectiveness must be commended and encouraged. Dauchy, Dolbeault, and Reich [114] argued that a specific fit between types of psychosocial therapies that are effective with patients’ expressive capacities, psychosocial situations, and timing in their process of oncological care must be identified.

Despite a seemingly solid universal consensus among experts that psychosocial therapies synergistically augment psychiatric medication interventions’ effectiveness and the two are mutually supportive, the shortage of empirical evidence means that the jury is still out ([232, 233], also see [4, 17, 23, 114, 211, 213, 230], cf. [25]). Solid, large-scale, prospective trials are needed to investigate whether the therapeutic alliance is supported by psychosocial interventions and augments psychiatric medication interventions with end-stage cancer patients (also see [23, 150, 234], cf. [17, 25, 211]). This research should establish what aspects of psychotherapy are more or less beneficial, such as mutual trust, respect, sensitivity, coping, mechanisms/tools, and decreasing/eliminating maladaptive thinking. The literature contains increasing calls to use less intensive and less invasive data sources, including secondary administrative and medical records [235]. Regarding psychosocial education interventions, after a spate of studies culminating in a series of meta-analytic studies, research on this aspect of psychiatric interventions has dissipated—and this should not be where it ends as there is always more to learn.

#### Spirituality

Though spirituality and religion (religiosity) were not the province of this study, they may have positive influences on psychiatric interventions and end-stage cancer care that are worthy of informed commentary. Patients in end-stage cancer naturally experience multi-layered and inter-connected physical problems and emotional stressors, such as deterioration in quality of life, pain and increased sensitivity to pain, difficulty communicating, loss of control, physical disfiguration, burdens placed on others, costs of care, self-demoralization, feelings of worthlessness, helplessness, guilt, indifference, loss of interest and pleasure, and pathological pessimism ([73], also see [25, 114], cf. [236]). End-stage cancer patients often confront existential questions such as: Why? Why me? What have I done to deserve this? They often need to find meaning in their suffering, death, and the afterlife, and their illness may cause them to draw on or seek refuge in their faith or religion as a way of coping. Recently, small yet promising trials testing the effectiveness of interventions designed to boost spirituality have found that spirituality can positively affect end-stage cancer patients’ mood and quality of life [237–241]. Not surprisingly, these studies found that spirituality interventions increased spirituality among the participants receiving these interventions. The good news is that some of the studies [212] are in the process of being replicated at several sites worldwide.

## Discussion

**Fig. 3:**
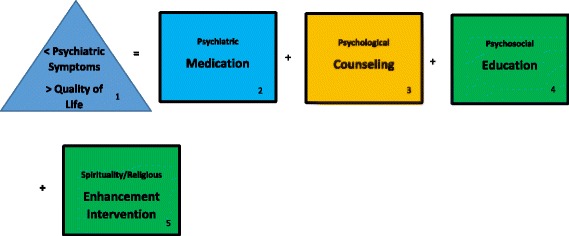
End-stage cancer patient psychiatric treatment effectiveness linear model—the therapeutic alliance

In sum, at the risk of over-simplification, this review of the literature revealed a testable linear model of a combination of psychiatric treatments for communicative and cognizant end-stage cancer patients, as shown in Fig. 3.

This model has correlative models that emerged through rigorous scientific retrospective study of the cost-effectiveness of treatments on other psychiatric patient populations [242] (Note: The weight or importance of co-factors is displayed by their order of precedence, left to right.) First, consistent with best practice guidelines, the goal of psychiatric treatments with end-stage cancer patients is reduction in their psychiatric symptoms and enhancement of their quality of life ([243], also see, [1, 2, 11, 49, 52, 152, 153]). Second, depending on symptoms, medications are the most powerful tools in the psychiatric arsenal and this armory is expanding and extending with new editions and off-label combinations (cf. [74]). Third, some form of psychological counseling/intervention appears to support and augment psychiatric medication interventions. Though their effectiveness might be mixed, common sense dictates that the potential harm is relatively minimal, and they can serve as vehicles for fortifying the therapeutic alliance. Fourth, though a connection between psychosocial education and psychiatric therapeutic effectiveness has yet to be established, such education has some beneficial or helpful effects for patients in crisis and research on it should not be abandoned entirely. Fifth, studies on interventions designed to enhance spirituality have shown substantially promising results in terms of quality of life and should be considered for inclusion in the constellation of future psychiatric treatment planning.

Of course, these recommendations take into account that the first priority is always treating physical medical conditions ([243], also see [7, 17, 27, 58, 74, 183, 211], cf. [22, 53, 114]). In terms of end-stage cancer patients who are cognitively impaired or unable to communicate (e.g., advanced delirium), psychiatric medication is probably the only appropriate therapeutic choice [74], but counseling, psychosocial education, and spirituality interventions may be appropriate interventions for family members and caregivers (cf. [25, 27, 73, 114]). Nevertheless, “the devil is in the details.” Specifically, the complicated and tricky part of each component in the model is negotiating, balancing, conforming, and/or fitting the right treatment with the right patient, diagnosis (cf. [5, 7, 27, 56, 58]), dose for response, titration and tapering, drug-drug interactions, route of administration, and context and doing so decisively and quickly. Though varying amounts of quality scientific evidence exists in terms of components in the model, the review reported herein noted that some studies lack supporting evidence, or worse offer pseudo-evidence, and that much more rigorous and larger scale scientific studies must be conducted. This is particularly true for research on end-stage cancer patients in general and newly developed treatments and off-label combinations in particular—and of course effectiveness studies that include psychiatric intervention costs (cf. [74, 242]).

As cancer treatments become more aggressive and successful, and as the populations of older adults and, by extension, the populations with end-stage cancer, grow, patients’ quality of life is of paramount importance. Delirium, clinical depression, and anxiety/adjustment disorder are complex and multi-faceted disorders and the psychotherapeutic and psychopharmacological interventions for their treatment require further study to inform more precise and powerful cancer care and best practices, especially in end-stage cancer. Thus, quality of life can be improved for patients dying of cancer. Universally endorsed care principles for end-stage cancer care affirm the importance of psychological health and access to the best mental health care that can be rendered to improve patients’ quality of life at life’s end ([244], also see [49, 52, 152, 153]). The wide dissemination of findings should alert clinicians and patients and their families as developments emerge. If anything, this article serves as an impassioned plea for further, better, and accelerated study of treatment effectiveness and care improvement for end-stage cancer patients’ psychiatric conditions, if only because these patients cannot wait.

## Conclusion

This article reported on a systematic, comprehensive, integrated review that investigated the effectiveness of interventions employed to treat psychiatric conditions common to end-stage cancer patients. The aim of this effort was to provide empirical findings to mental health practitioners so that they can hone their expertise in reducing emotional distress and psychiatric discomfort common to actively dying cancer patients and help these patients cope and improve their overall quality of life. Also, this work attempted to achieve an understanding of the state of the research on which best practices is predicated and to shed light on avenues for further inquiry. Undoubtedly, based on this review, more effective interventions are needed for psychiatric conditions/disorders; also needed are rapid and effective treatments to relieve psychiatric symptoms and disorders as well as strategies and tools to reduce or prevent them in advance [27].

## Abbreviations

<: Less than
**>**: Greater than
BDZ: Bio-diazepam
CBT: Cognitive behavioral therapy
ECT: Electroconvulsive shock therapy
LTC: Lieutenant colonel
MAO: Monoamine oxidase inhibitor
PTSD: Post traumatic stress disorder
SNRI: Serotonin–norepinephrine reuptake inhibitors
SSRI: Selective serotonin reuptake inhibitors
TCA: Tricyclic antidepressants
TMC: Texas medical center
UT-MDACC: University of Texas MD Anderson cancer center
